# The multifaceted role of AIF-1 in metabolic dysregulation: bridging inflammation, insulin resistance, and obesity

**DOI:** 10.3389/fendo.2026.1735573

**Published:** 2026-01-30

**Authors:** Jie Huang, Fei Jiang, Yinling Chen

**Affiliations:** 1 School of Medicine, Hangzhou City University, Hangzhou, China; 2 Anji People’s Hospital, Affiliated Anji Hospital, School of Medicine, Hangzhou City University, Huzhou, China

**Keywords:** adipocyte, allograft inflammatory factor-1, inflammation, insulin resistance, obesity

## Abstract

Allograft inflammatory factor-1 (AIF-1), a cytokine secreted by activated monocytes, macrophages, and lymphocytes, has emerged as a critical regulator of pathological processes spanning renal diseases, rheumatoid arthritis, cancer, cardiovascular disorders, neurological pathologies, and transplant-related conditions. Population-based studies have associated sequence variants near the AIF-1 locus with obesity, though AIF-1’s potential pathophysiological involvement remains uninvestigated. Understanding its molecular characteristics, receptor interactions, and signaling pathways is essential for elucidating its biological functions. This review comprehensively examines AIF-1’s involvement in inflammatory and metabolic pathogenesis, particularly focusing on obesity and inflammation. Through systematic literature analysis, we consolidated current knowledge on AIF-1’s functions and analyzed studies exploring its roles in obesity, insulin resistance, and inflammation to clarify broader disease mechanisms. AIF-1 exerts pleiotropic effects on immune cells, insulin signaling, and adipocytes. Elevated AIF-1 levels correlate with inflammatory adipocytes and obesity, while reduced AIF-1 promotes weight loss through regulation of monoamine oxidase A and decreased leptin/resistin production. Deciphering AIF-1’s complex roles in inflammation and metabolic disorders offers critical insights for therapeutic development. Targeting AIF-1 or AIF1-like (AIF1L) may yield novel strategies to mitigate disease progression and enhance clinical management of obesity.

## Graphical Abstract

**Figure:**
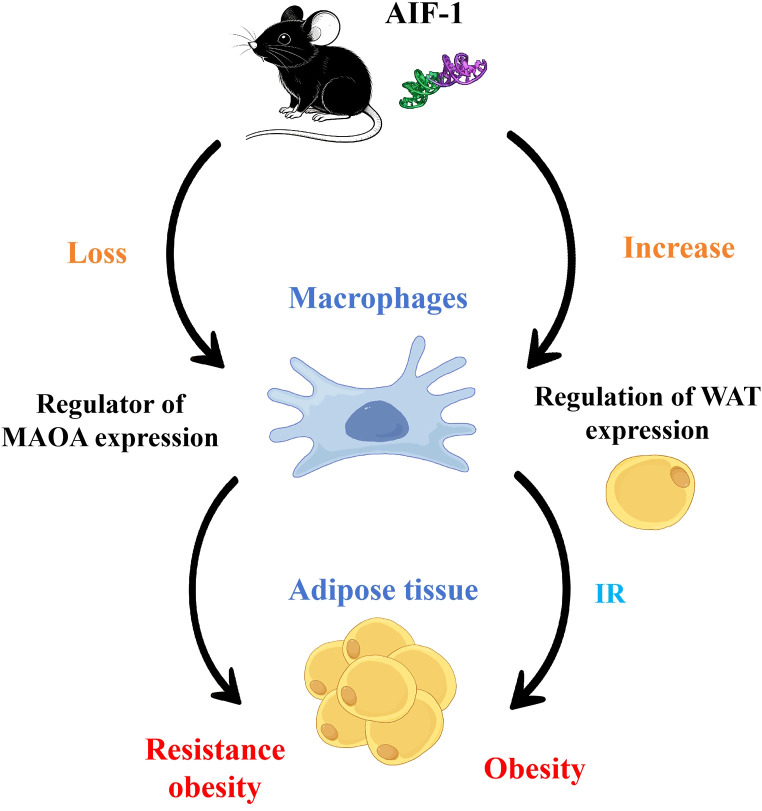
The dual role of AIF-1 in obesity. Enhanced AIF-1 mediates obesity. Loss of AIF-1 induces obesity resistance. AIF-1, allograft inflammatory factor-1; MAOA, monoamine oxidase a; WAT, white adipose tissue; IR, insulin resistance.

## 1 Introduction

AIF-1, an inflammatory factor first identified in rat cardiac graft chronic rejection (1, 2), with particular emphasis on its emerging roles in metabolic dysregulation. Originally implicated in xenograft rejection and macrophage activation (3), AIF-1’s pleiotropic functions now encompass rheumatoid arthritis, fibrotic, cardiovascular, neoplastic, and renal pathologies through multifaceted mechanisms (4–7). Crucially, its actin crosslinking capacity enables cytoskeletal remodeling that fundamentally alters cellular kinematics (8, 9), establishing mechanistic connections to systemic metabolic processes.

**Figure 1:**
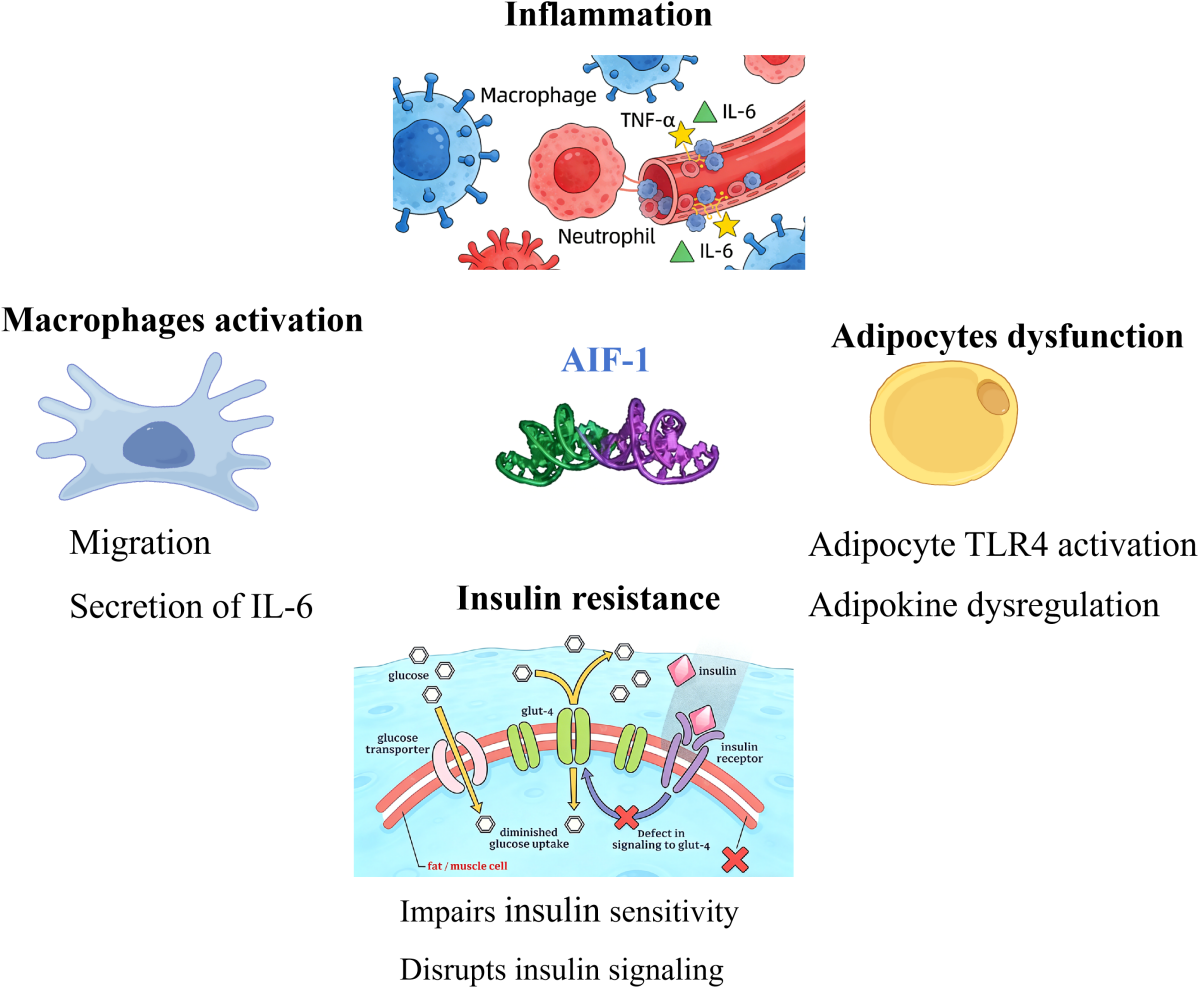
Overview of AIF-1 functions. Activation of macrophages, inducing insulin resistance, adipocytes dysfunction, and activation of inflammation. AIF-1, allograft inflammatory factor-1; IL-6: interlukin 6; TNF-α : tumor necrosis factor α; TLR-4: Toll-like receptor-4.

Currently, AIF-1 is extensively utilized in function researching (**Figure 1**). Its involvement in allogeneic transplantation responses, regulation of immune responses, multifunctional cytokine roles, and promotion of cell proliferation offer valuable targets and insights for exploring disease mechanisms and developing therapies (2). Some studies offer evidence linking AIF-1 to obesity, although the mechanism of action is sitll unclear. At first, a single nucleotide polymorphism research showed AIF-1 gene is associated with body weight (10). Additionally, loss of AIF-1 limits diet induced obesity and insulin resistance (11). Furthermore, AIF1-like (AIF1L) affects food intake and obesity, especially for high fat diet (HFD) induced obesity (12).

On the other hand, AIF-1 is a portein that regulates the function of macrophages and refers to inflammation response (13). Besides, AIF-1 is also as a calcium-banding protein participants in activation of macrophages (14). Meanwhile, AIF-1 may be a new adipokine associated with adipose inflammation in obese individuals (15).

Of particular significance, AIF-1 directly interacts with adipocytes to coordinate inflammatory signaling and insulin pathway modulation (7), mechanistically explaining its capacity to induce insulin resistance through multi-target disruption (15) and oxidative stress potentiation (16).

In this review, we focus on the function of AIF-1, especially in obesity, inflammation, macrophages and adipocytes dysfunction, and insulin resistance. This synthesis evaluates current evidence while proposing novel research trajectories, ultimately aiming to translate mechanistic insights into targeted therapeutic strategies for metabolic disorders.

## 2 Methodology

### 2.1 Search strategies

**Figure 2:**
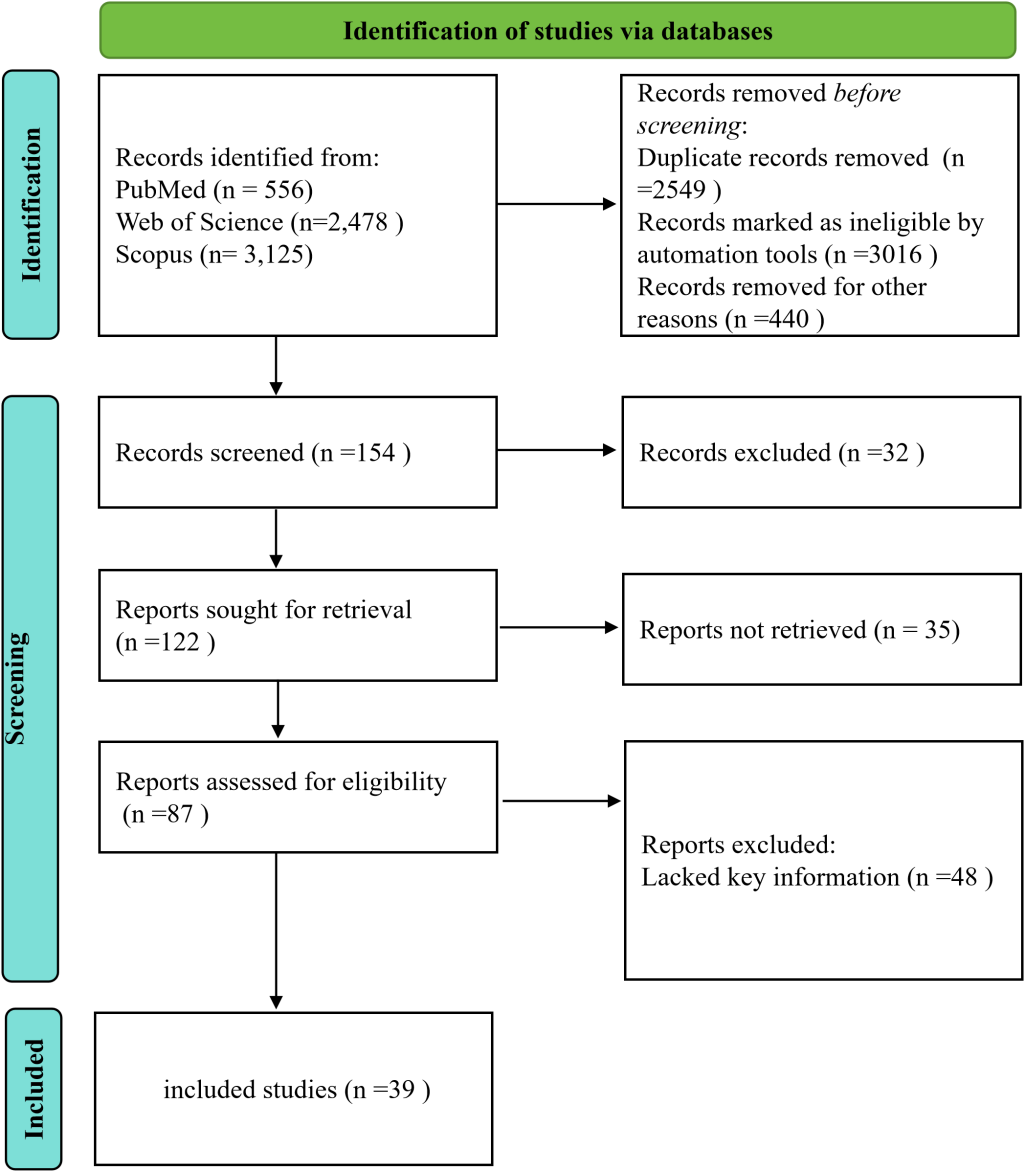
PRISMA flow chart.

The literature search supporting this review employed a targeted exploration of biomedical databases including PubMed, Web of Science, and Scopus (**Figure 2**). Relevant studies were searched from the databases from inception until December 2025. A combination of controlled vocabulary terms (MeSH headings) and free-text keywords such as “AIF-1” or “Iba1”, “obesity” or “obese”, “insulin resistance” or “IR”, “adipocyte” or “adipose tissue”, and “inflammation” or “inflammatory”, were iteratively applied. The search strategy encompassed all publication types without language restrictions, though non-English publications were retained only if accompanied by English abstracts. Retrieved articles were filtered to eliminate duplicates and irrelevant results. Additionally, the reference lists of selected articles were reviewed to identify other pertinent publications.

### 2.2 Inclusion and exclusion criteria

Included criteria: (1) The results of the researches should include body weight, AIF-1 level, adipose tissues, or inflammatory response; (2) The research subjects are animals or humans; (3) Relevant review for AIF-1. Exclusion criteria: (1) Personal experience summaries; (2) Studies displayed as figures; (3) Inconsistent with the inclusion criteria.

## 3 Function in immunity: macrophages

AIF-1 has been reported to play a significant role in immune response and inflammatory pathology. Immunohistochemical studies have shown that AIF1 is present in all macrophage subpopulations except for alveolar macrophages and sperm cells (17). Considering the role in macrophages, AIF-1 was indicated to be crucial for cell survival and proinflammatory activities (18). *In vitro*, studies reported that AIF-1 level increase promoted macrophages cell migration, but downregulation of AIF-1 reduced nitric oxide (NO) production and led to macrophages apoptosis (18). In published papers, overxpressed AIF-1 in the macrophage cell line (RAW 264.7) produced a large number of inflammatory factor, such as interlukin (IL)-6 and IL-10 (19). Furthermore, inhibiting expression of AIF-1 resulted in a decrease in migration, proliferation, and signal transduction of Akt and MAPK signal pathway (20).

## 4 Function in adipose tissue: adipocyte

AIF-1 engages in multifaceted crosstalk with adipocytes through inflammatory and metabolic regulatory axes. Emerging evidence implicates TLR4/MD2 complexes as primary AIF-1 receptors in adipocytes, with binding initiating NF-κB and JNK signaling cascades that drive inflammatory responses (21, 22). This receptor-mediated activation establishes a feedforward loop between macrophage-derived AIF-1 and adipocyte dysfunction.

### 4.1 Inflammatory crosstalk in adipocytes

Obesity-induced adipose inflammation triggers macrophage AIF-1 overproduction, which via paracrine signaling enhances adipocyte TLR4 activation (15, 23). This interaction stimulates IL-6 and TNF-α secretion while suppressing adiponectin, creating a self-sustaining inflammatory milieu (24). Crucially, AIF-1 knockdown models demonstrate reduction in macrophage infiltration and decrease in leptin/resistin production (11), mechanistically linking AIF-1 to adipokine dysregulation.

### 4.2 Adipokine secretion modulation

AIF-1 directly reprograms adipokine secretion profiles through TLR4-dependent signaling. Stimulation of adipocytes with recombinant AIF-1 (100 ng/mL) elevates TNF-α and IL-6 secretion, while suppressing adiponectin production (15, 25). This bidirectional regulation mechanistically involves JNK/STAT3 dual-pathway activation: phosphorylation analyses reveal increased STAT3(Y705) and elevated JNK(T183/Y185) upon AIF-1 exposure (22). The resultant proinflammatory shift (TNF-α/adiponectin ratio increased) establishes a self-reinforcing metabolic-inflammatory loop that perpetuates insulin resistance (26). Critically, AIF-1-induced adipokine dysregulation exhibits dose-dependency (EC50 = 38 nM) and correlates with impaired GLUT4 trafficking, directly linking inflammatory signaling to metabolic dysfunction.

## 5 Potential interactions with inflammatory cytokines

AIF-1 is a protein that induces inflammatory responses and macrophages migration (14). Previous studies indicated taht AIF-1 promotes inflammatory cytokines releasion in mice macrophages (27, 28). AIF-1 promoted vascular smooth muscle cells dedifferentiation into macrophage-like state, enhancing the production of inflammatory cytokines TNF-α and IL-6 by PKC/NF-κB pathway (29). Furthermore, Loss of AIF-1 femal mouse showed increased expression of pro-inflammatory cytokines, such as TNF-α and IL-6. Then, aggravation of cardiac inflammation (30). However, AIF-1 regulation induced the releasion of inflammatory cytokines TNF-α and IL-6 in diabetic mouse model (31). The differences in the results might be due to the different subjects of the research.

An adipocyte-secreted polypeptide, adiponectin, playing a key role in the inhibition of metabolic derangements (32). In contrast to adiponectin, the level of resistin was increased in individuals with IR (33). Meanwhile, the study demonstrated that AIF-1 increased the resistin production, while reduced adiponectin secretion from 3T3L1 adipocytes probably through NF-κB pathway activation, and inhibiting PPARγ level (16).

## 6 Metabolic disease: obesity

Some studies indicated AIF-1 was associated with metabolic conditions in different subjects (**Table 1**). First, the serum AIF-1 levels linked to waist circumference in Japanese (21). Second, sequence variants near the AIF-1 gene locus are correlated with adult obesity in Greeks (35). Third, a recent study shows AIF-1secreted by macrophages in white adipose tissue for obese female (15). Additionally, *in vivo* studies reported that loss of AIF-1 limited HFD induced obesity and diabetes (11). Furthermore, AIFIL could accelerated HFD-induced obesity in some settings (12). However, one studies showed that loss of AIF1L did not affect HFD-induced weight gain or recover glucose sensitivity (34). The reasons for the differences in results may be as follows: (1) different genetic backgrounds and experimental models; and (2) discrepancies in dietary protocols or metabolic stress contexts for mouse.

**Table 1:** Overview of the association between AIF-1/AIF1L and obesity.

Subject	AIF-1/AIF1L expression	Type of expressing cell	Signaling pathway	Mechanism of action	References
Obese women	Increased	Macrophages of WAT	Adipokines in adipose tissue	Inhibiting production of adiponectin	(15)
Transgenic mice	Loss	All types of cells	A genetic modifier of *Obrq2* and leptin	Increasing leptin sensitivity, regulating leptin levels.	(12)
Mice	Loss	All types of cells	An AIF1–MAOA regulating axis	Affecting NE catabolism in macrophages, it may contribute to obesity and associated insulin resistance and glucose intolerance	(11)
Mouse	Loss	All types of cells	No effect on insulin sensitivity or HFD-induced glucose insensitivity	No differences in fat or lean mass accumulation, and displayed no changes in energy expenditure or systemic glucose handling.	(34)

WAT, white adipose tissue; MAOA, monoamine oxidase a; NE, norepinephrine; HFD, high fat diet.

Although evidence suggests that AIF-1 is related to obesity, the specific mechanism is still less clear. The existing literature indicates relationship between AIF-1 and obesity focus on inflammatory mediation—a hallmark of obesity-associated metabolic dysfunction (36, 37). As a key driver of localized and systemic insulin resistance, inflammation is profoundly regulated by AIF-1, predominantly secreted by adipose tissue macrophages (38). Mechanistically, AIF-1 modulates macrophage catecholamine activity to suppress energy expenditure while enhancing storage capacity, thereby promoting adiposity (11). During obesity progression, adipose-resident macrophages undergo functional polarization from anti-inflammatory to pro-inflammatory phenotypes, driving tissue infiltration and inflammatory exacerbation (39). This phenotypic shift correlates with macrophage-derived pro-inflammatory mediator production, wherein AIF-1 emerges as a critical molecular orchestrator (10, 16).

**Figure 3:**
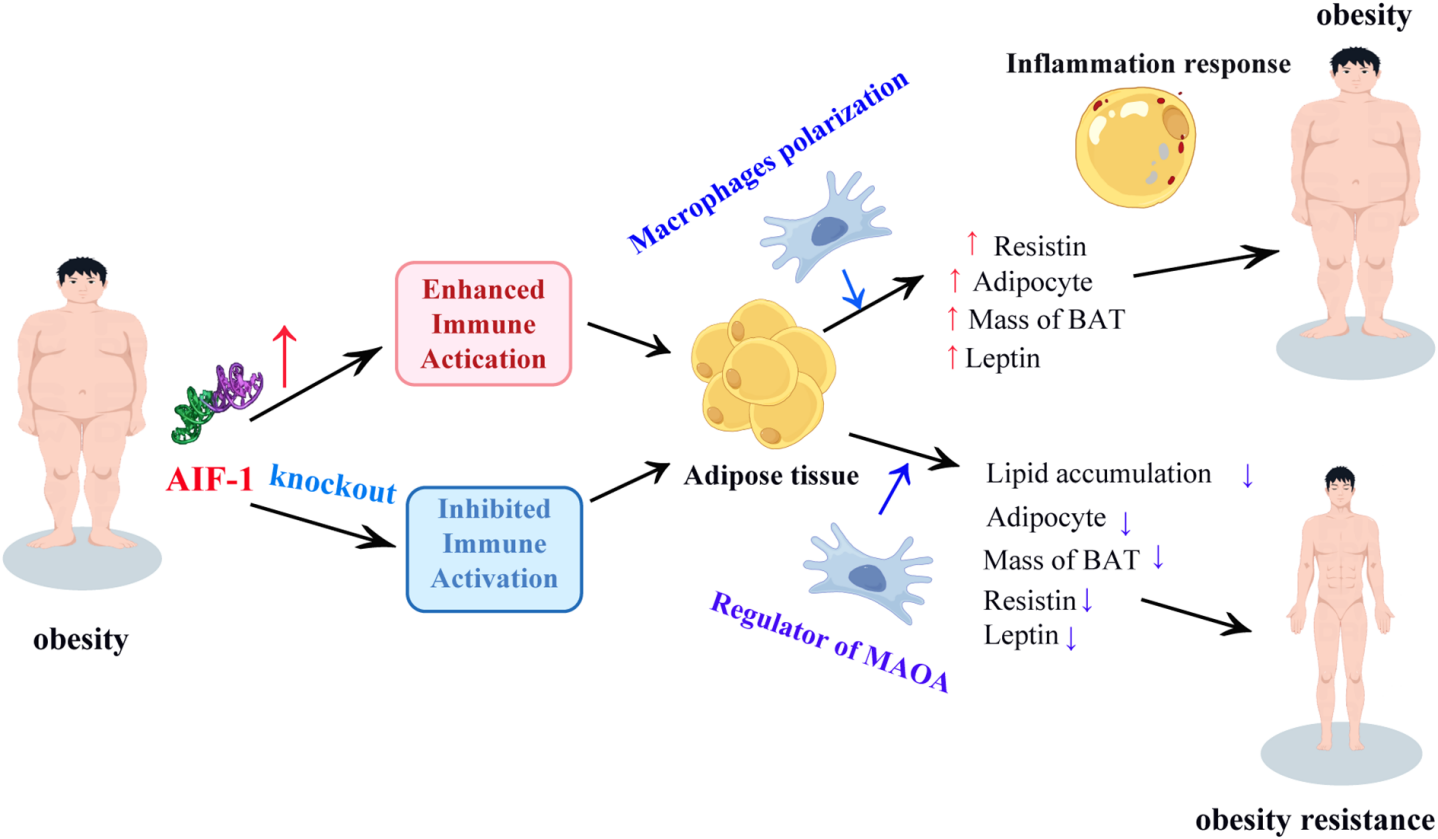
Regulation of adipocyte function and inflammatory signaling in obesity. An increase in AIF-1 levels stimulates the polarization of inflammatory-induced adipose tissue macrophages, increases leptin and resistin levels, increases fat cell mass, and enhances the inflammatory response leading to obesity. On the contary, loss of AIF-1 inhibited inflammation response to regulate MAOA expression in macrophages with decreased lipid accumulation, mass of adipocytes, BAT, and decreased leptin and resistin inducing obesity resistance. AIF-1, allograft inflammatory factor-1; MAOA, monoamine oxidase a; BAT, brown adipose tissue.

Clinical evidence demonstrates elevated AIF-1 expression in obese individuals, particularly within adipose depots (10, 40). This upregulation likely originates from obesity-associated chronic inflammation (22), with heightened AIF-1 levels correlating with increased obesity susceptibility (23). Importantly, studies have shown that decreased AIF-1 levels inhibit adipocyte differentiation and reduce the secretion of adipokines such as leptin and resistin, which are critical mediators of metabolic dysfunction in obesity (15, 16, 41). For instance, Lorente-Cebrián et al. demonstrated that AIF-1 knockdown in adipocytes led to a significant reduction in leptin and resistin expression, highlighting its role in regulating adipokine production (15). Similarly, Ren et al. reported that AIF-1 deficiency in macrophages attenuated pro-inflammatory cytokine release and improved insulin sensitivity in obese mice (16). These findings collectively support the notion that AIF-1 might be a key regulator of adipocyte function and inflammatory signaling in obesity (**Figure 3**).

### 6.1 Adipocytes

Emerging evidence suggests AIF-1 mediates paracrine regulation of adipose functionality (42) through intricate macrophage-adipocyte crosstalk (15). Such cellular interactions profoundly disrupt metabolic coordination, directly fueling obesity-associated pathological cascades. Study showed that AIF-1 stimulates the production of reactive oxygen species (ROS) in adipocytes by elevating ROS production through NOX4 upregulation, exacerbating metabolic dysfunction (36). Additionally, acute AIF-1 exposure (48h) paradoxically enhances lipid droplet formation in differentiating adipocytes (16), while chronic exposure (28 days) reduces lipolytic capacity through PPARγ suppression. This temporal dichotomy mirrors clinical observations of AIF-1 overexpression in obese patients correlating with increased ectopic lipid deposition (43), impairment in insulin signaling fidelity, and elevated cardiovascular risk markers. Mechanistically linking these effects, AIF-1 activates mTORC1-SREBP1 cascades (phosphorylation increased) that reprogram lipid metabolism while simultaneously inhibiting AMPKα (Thr172) phosphorylation (16). Beyond its inflammatory mediation, AIF-1 emerges as a multifunctional hub integrating molecular networks that drive both obesity initiation and its metabolic sequelae, positioning it as a critical node in adiposopathy pathogenesis.

### 6.2 Inflammation

Adipose tissue macrophages are the main sources of the proinflammatory molecules, such as TNF-α and IL-6 (38). AIF-1 operates as a key pro-inflammatory effector in adipose microenvironments (20), orchestrating macrophage activation/recruitment and immune cell infiltration that establishes a pathological feedback loop of chronic inflammation and insulin resistance (44). This self-perpetuating cascade perpetuates tissue damage while destabilizing systemic metabolic homeostasis. Clinically, elevated AIF-1 expression in obesity correlates with detrimental metabolic manifestations-including pronounced insulin resistance and dyslipidemia (45) -which reciprocally amplify inflammatory signaling to accelerate adiposity progression and comorbidity development.

### 6.3 Insulin signaling disruption

**Figure 4:**
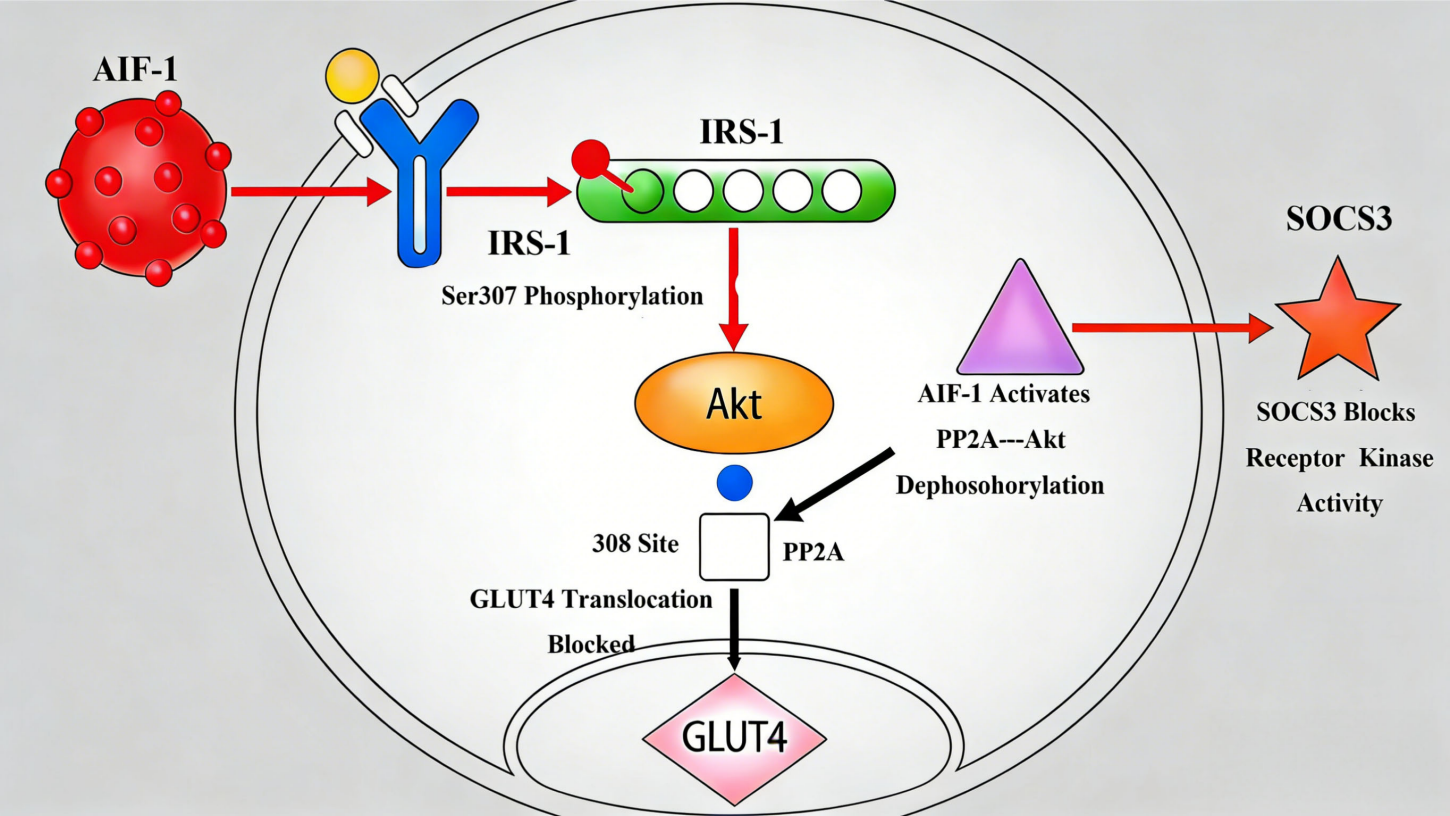
AIF-1 disrupts insulin signal pathway in 3T3-L1 adipocytes. Phosphorylating IRS-1 at Ser307, impairing insulin receptor substrate function, downregulating GLUT4 translocation through PI3K/Akt pathway inhibition, and inducing SOCS3 expression, which blocks insulin receptor tyrosine kinase activity resulted in disorder of insulin signal pathway. AIF-1, allograft inflammatory factor-1; IRS-1, insulin receptor substrate 1; GLUT4, glucose transporter 4; SOCS3, suppressor of cytokine signaling 3.

The emerging role of AIF-1 in metabolic regulation is particularly evident in obesity-associated pathologies, where chronic adipose tissue inflammation serves as a critical nexus linking AIF-1 dysregulation to insulin resistance development (46). AIF-1 impairs insulin sensitivity through multi-tiered mechanisms. *In vitro* studies showed that AIF-1 disrupts insulin signaling in 3T3-L1 adipocytes by phosphorylating IRS-1 at Ser307, impairing insulin receptor substrate function, downregulating GLUT4 translocation through PI3K/Akt pathway inhibition, and inducing SOCS3 expression, which blocks insulin receptor tyrosine kinase activity (47) (**Figure 4**). Additionally, AIF-1 may disrupt insulin signaling by suppressing Akt pathway with decreasing Akt308 phosphorylation by PP2A activation (37). Besides, AIF-1 impairs insulin signaling via upregulating the release of inflammatory factors (TNF-α, IL-6, and resistin) and downregulating the secretion of insulin-sensitive factors (adiponectin) (19, 24). Moreover, AIF-1 knockout models demonstrated improved insulin sensitivity (HOMA-IR reduction), enhanced glucose tolerance (AUC decrease), and reduced adipose tissue macrophage infiltration (48). Furthermore, clinical studies elucidated that type 1 diabetic patients show altered AIF-1-mediated immune regulation with reduction in pancreatic islet T-cell populations, downregulated IFN-γ/T-bet axis in AIF-1-silenced individuals, and compensatory expansion of CD25+Foxp3+CD4+ Treg cells (49). These coordinated effects establish AIF-1 as a master regulator of adipocyte insulin resistance, with preclinical studies showing AIF-1 inhibition restores normal insulin response (50). The conserved AIF-1/insulin resistance axis across species (from *in vitro* models to human pathophysiology) underscores its potential as a therapeutic target.

## 7 Therapeutic potential and translational challenges

Inhibiting AIF-1 presents a compelling therapeutic strategy for combating obesity and its metabolic complications. Preclinical studies demonstrate that AIF-1 deficiency protects mice from diet-induced obesity by elevating local catecholamine levels in adipose tissue, reducing macrophage expression of the degradation enzyme MAOA. This enhances noradrenergic signaling, stimulates energy expenditure, and promotes a thermogenic phenotype. Concurrently, AIF-1 drives adipose tissue inflammation and directly impairs insulin signaling in adipocytes. Human genetic studies and analyses of adipose tissue samples consistently link elevated AIF-1 expression to obesity and insulin resistance, suggesting conserved biological relevance. However, while these human associative data are promising, critical discussion must note the current lack of robust clinical data validating AIF-1 as a reliable diagnostic biomarker or a therapeutically actionable target in human obesity. Its potential hinges on these compelling preclinical and correlative human findings, which await validation through prospective clinical trials.

However, translating this promising target into a safe and effective therapy is fraught with significant challenges, which are further compounded by the limited human clinical evidence. The primary risk of serious off-target effects arises because AIF-1 is not adipose-specific; it is a key functional protein in immune cells throughout the body and serves critical roles in brain microglia. Systemic inhibition could therefore disrupt central nervous system function and compromise innate immunity. Furthermore, the precise molecular pathway by which AIF-1 regulates MAOA expression remains unclear, and potential gender-specific responses add complexity. The absence of clinical trial data makes it difficult to predict efficacy, optimal dosing, or long-term safety in humans. Overcoming these hurdles will require not only the development of highly targeted delivery systems to confine therapeutic action to relevant adipose tissue macrophages but also a substantial investment in clinical research to bridge the gap between promising mechanistic biology and validated human therapeutic application.

## 8 Conclusions

**Figure 5:**
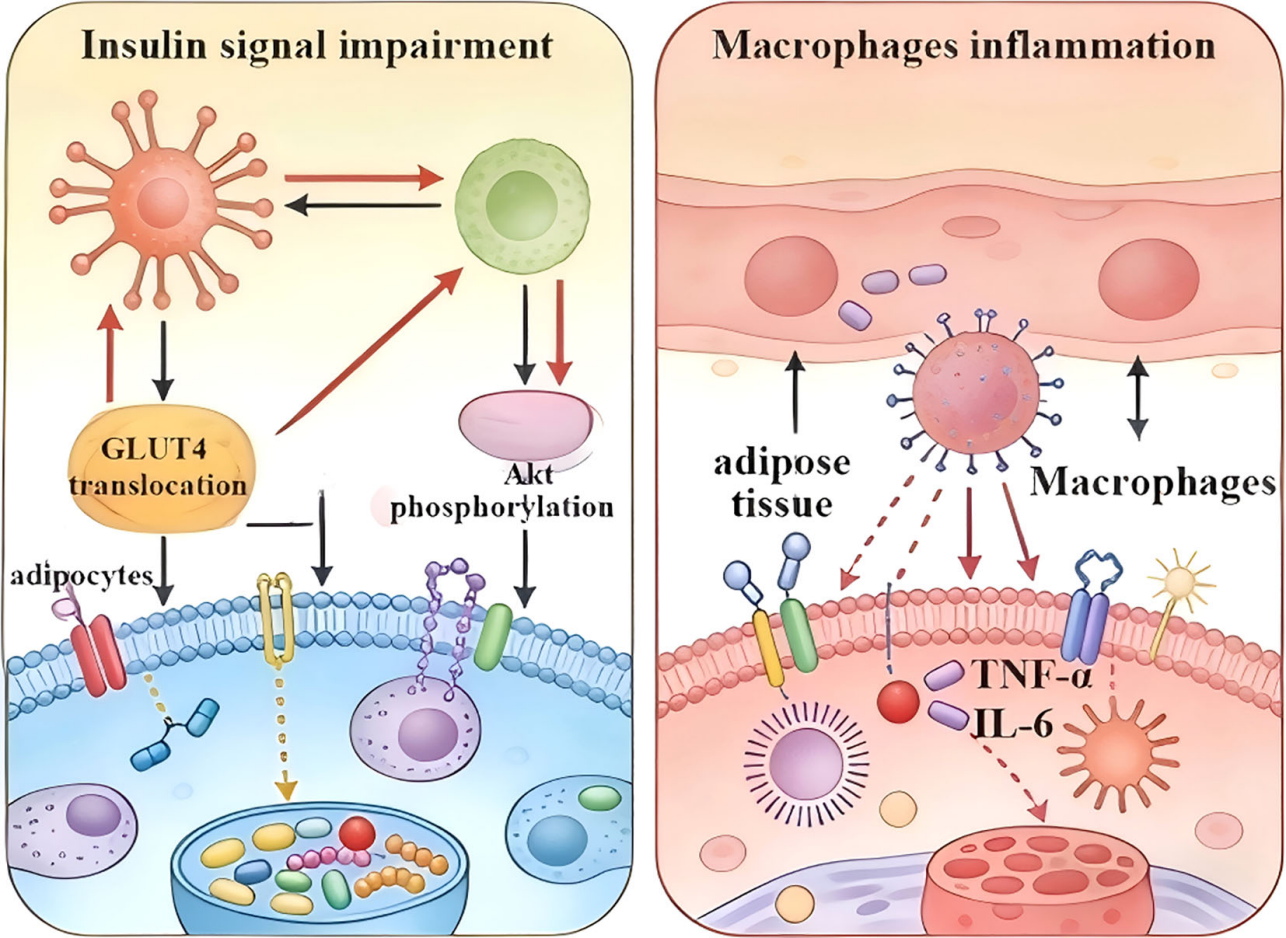
AIF-1 regulates insulin signaling and macrophage inflammation. Macrophage-derived AIF-1 secretion adipokine TNFα, and IL-6. Moreover, it suppressed insulin-stimulated glucose uptake by down-regulating insulin signaling with GLUT4 translocation, and Akt phosphorylation. AIF-1, allograft inflammatory factor-1; TNF-α, tumor necrosis factor; IL-6, interlukin 6; GLUT4, glucose transporter 4.

In summary, AIF-1 has emerged as a critical and multifaceted regulator at the intersection of inflammation and metabolism, presenting a novel and compelling target for the management of obesity (**Figure 5**). The pathophysiology of AIF-1 may include following: (1) metabolic dysregulation: orchestrating adipose tissue inflammation through macrophage; (2) insulin signaling interference: impairing GLUT4 translocation and Akt phosphorylation induced insulin signal impairment in adipocyte; and (3) inflammation activation: stimulating inflammatory cytokines releasion in macrophage for diet-induced obesity.

However, the translation of these mechanistic insights into clinical applications faces challenges. Population genetics and human adipose tissue analyses strongly associate AIF-1 with obesity, but robust clinical data validating it as a diagnostic biomarker or a directly druggable target remain a critical gap. The primary translational challenge lies in the precise targeting of AIF-1’s pathogenic actions without incurring off-target effects, given its role in general immunity. Future strategies may involve developing tissue-selective delivery systems or exploiting the nuanced biology of its paralog, AIF1L. Besides, for translational potential, we suggest to interrupte the effect of AIF-1 on gene expression as an effective way to alleviate obesity.
